# Antimicrobial Activity of *Lactobacillus* Species Against Carbapenem-Resistant *Enterobacteriaceae*

**DOI:** 10.3389/fmicb.2019.00789

**Published:** 2019-04-18

**Authors:** Chi-Chung Chen, Chih-Cheng Lai, Hui-Ling Huang, Wen-Yu Huang, Han-Siong Toh, Tzu-Chieh Weng, Yin-Ching Chuang, Ying-Chen Lu, Hung-Jen Tang

**Affiliations:** ^1^Department of Medical Research, Chi Mei Medical Center, Tainan, Taiwan; ^2^Department of Food Science, National Chiayi University, Chiayi, Taiwan; ^3^Department of Intensive Care Medicine, Chi Mei Hospital, Tainan, Taiwan; ^4^Department of Health and Nutrition, Chia Nan University of Pharmacy and Science, Tainan, Taiwan; ^5^Department of Intensive Care Medicine, Chi Mei Medical Center, Tainan, Taiwan; ^6^Department of Medicine, Chi Mei Medical Center, Tainan, Taiwan

**Keywords:** *Lactobacillus*, carbapenem-resistant, *Enterobacteriaceae*, *in vitro* – antibacterial, activity

## Abstract

**Objective:**

This study aims to identify suitable lactobacilli that have anti-carbapenem-resistant *Enterobacteriaceae* (CRE) activity with *in vitro* tolerance to pepsin and bile salts.

**Methods:**

Fifty-seven *Lactobacillus* spp. strains encompassing nine species were collected for investigation. Their viabilities in the presence of pepsin and bile salts were tested using tolerance tests. Their anti-CRE effects were assessed by agar well diffusion and broth microdilution assay, as well as time-kill test.

**Results:**

Of the 57 *Lactobacillus* isolates collected, 31 had a less than 2-log reduction in their viability in both pepsin and bile salt tolerance tests. Of these 31 isolates, 5 (LUC0180, LUC0219, LYC0289, LYC0413, and LYC1031) displayed the greatest anti-CRE activity with a CRE zone of inhibition greater than 15 mm in agar well diffusion assays. The minimal inhibitory percentages of supernatants from these five strains against CREs ranged from 10 to 30%. With the exception of LUC0180, which had a minimal bactericidal percentage ≥ 40%, the bactericidal percentage of all the strains ranged from 20 to 40%. The inhibitory effect of the cell-free culture supernatants from these *Lactobacillus* strains did not change after heating but was abolished as the pH changed to 7.0. After a 24-h incubation, five of the *Lactobacillus* strains at a concentration of 10^8^ CFU/ml totally inhibited the growth of carbapenem-resistant *Escherichia coli* (CRE316) and *Klebsiella pneumoniae* (CRE632). After a 48-h incubation, the growth of CRE316 was completely inhibited under each concentration of lactobacilli based on time-kill test. Furthermore, when the concentration of lactobacilli was at 10^8^ CFU/ml, the decline in pH was faster than at other concentrations.

**Conclusion:**

Some *Lactobacillus* strains exhibit anti-CRE activity, which suggests potential applications for controlling or preventing CRE colonization or infection.

## Introduction

Although *Enterobacteriaceae* are normal flora of the human intestinal system, they are also common pathogens causing human infections in the setting of both community-acquired and healthcare-associated infections (Hsueh et al., 2010; Toh et al., 2012; Lai et al., 2014; Jean et al., 2016). In this era of widespread antibiotic resistance, *Enterobacteriaceae* are no exception. Recently, the emergence of carbapenem-resistant *Enterobacteriaceae* (CRE) has become a more critical issue due to the limited therapeutic options available for these pathogens and the significant morbidity and mortality associated with CRE infections (Tang et al., 2016a; Rodriguez-Bano et al., 2018). Therefore, there is an urgent need for new treatments for these critical CRE-associated conditions.

*Lactobacillus* is one of a number of probiotics considered to be biological therapeutics and host immune-modulating biologicals that are generally recognized as safe (GRAS). Recent studies demonstrated several antimicrobial mechanisms of *Lactobacillus* such as nutrient competition, production of inhibitory compounds, immune-stimulation and competition for binding sites. In addition, *Lactobacillus* can produce lactic acid, acetic acid, formic acid and other acids to reduce intestinal pH, which may be the most important mechanism. These bacteria can also secrete certain antimicrobial molecules, such as ethanol, fatty acid, hydrogen peroxide and bacteriocins to exert the antimicrobial activity (Georgieva et al., 2015; Inglin et al., 2015). Through these mechanisms, *Lactobacillus* has demonstrated its ability to inhibit several bacterial pathogens, including *Clostridium difficile* (McFarland, 2015), *Escherichia coli* (Kumar et al., 2016), *Shigella* spp. (Mirnejad et al., 2013), *Streptococcus mutans* (Ahn et al., 2018), *Pseudomonas aeruginosa* (Jamalifar et al., 2011), and *Staphylococcus aureus* (Kang et al., 2017). However, no previous studies have assessed the antimicrobial activity of *Lactobacillus* against CRE. Thus, we conducted this study to identify suitable lactobacilli that have anti-CRE activity with *in vitro* tolerance to pepsin and bile salts.

## Materials and Methods

### Bacterial Strains and Culture Conditions

Fifty-seven *Lactobacillus* spp. strains encompassing nine species were obtained from Department of Food Science at the National Chiayi University in Chiayi, Taiwan. Species confirmation was performed by 16S rDNA sequencing. A fragment of the 16S rDNA was amplified by PCR. After amplification, the amplicons were separated by gel electrophoresis and sequenced. Sequences were compared with the NCBI GenBank database using the BLAST search tool to find the closest matches. The basic growth media for LAB were Man-Rogosa-Sharpe (MRS; Oxoid Inc., Ogdensburg, NY, United States).

Twenty clinical strains, including 10 different pulse field gel electrophoresis (PFGE) genotyped carbapenem-resistant *Escherichia coli* (CREC) and *Klebsiella pneumoniae* (CRKP) strains were isolated from Chi Mei Medical Center (Lai et al., 2016; Tang et al., 2016b). Species confirmations were performed using the VITEK 2 automated system (bioMérieux, Marcy l’Etoile, France) with standard biochemical methods. Mueller Hinton (MH) broth (Difco Laboratories, Detroit, MI, United States) were used for bacterial pathogens. The isolates were stored at −80°C in Protect Bacterial Preservers (Technical Service Consultants Limited, Heywood, United Kingdom) before use.

Carbapenem susceptibility testing was performed using the disk diffusion method according to the Clinical and Laboratory Standards Institute (CLSI) guidelines (Clinical and Laboratory Standards Institute [CLSI], 2015). In brief, a 0.5 McFarland turbidity standard inoculum from overnight cultures was followed by incubation of Mueller-Hinton agar plates at 35°C. The antibiotic disks were placed on the agar surface. After 16–18 h of incubation at 35°C, results were interpreted as either sensitive, intermediate, or resistant according to the inhibitory zone diameters around the disks using CLSI breakpoints. Carbapenem resistance was defined as resistance to imipenem, meropenem, doripenem, or ertapenem.

### Pulsed-Field Gel Electrophoresis

**FIGURE 1:**
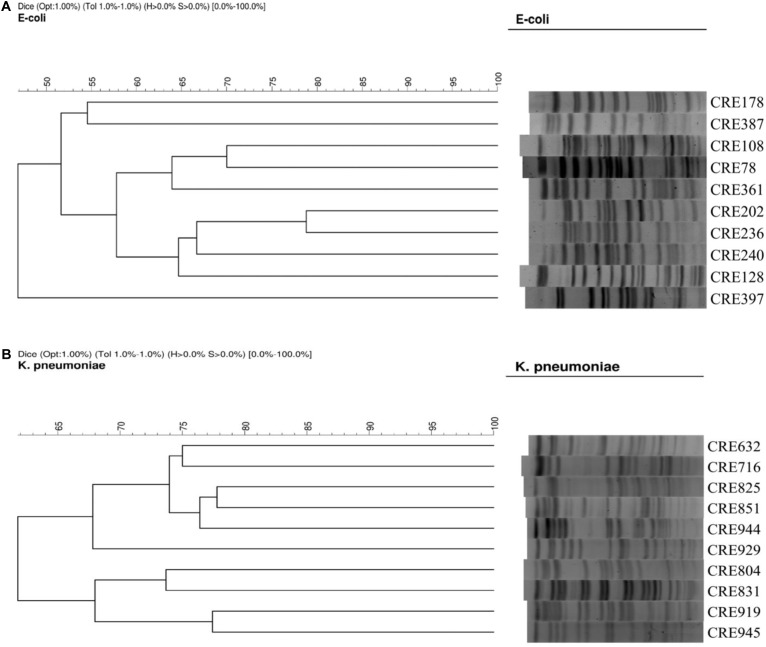
**(A)** PFGE of 10 isolates of carbapenem-resistant *E. coli.* **(B)** PFGE of 10 isolates of carbapenem-resistant *K. pneumoniae.*

Pulsed-field gel electrophoresis (PFGE) for the *Escherichia coli* and *K. pneumoniae* isolates were performed as described previously (Lai et al., 2016) with a CHEF DR II apparatus (Bio-Rad Laboratories, Hercules, CA, United States). Briefly, bacterial chromosomal DNAs were digested using XbaI (New England Biolabs, Beverly, MA, United States). Electrophoresis was carried out for 22 h at 14 μC, with pulse times ranging from 2 to 40 s at 6 V/cm, using a Bio-Rad CHEF MAPPER apparatus (Bio-Rad Laboratories, Richmond, CA, United States). A dendrogram based on the unweighted pair group was generated using methods previously described. The PFGE patterns were visually examined and interpreted according to the criteria of Tenover et al. (1995). The similarities of the PFGE profiles of each strain were compared using a Dice coefficient at 1.0% of tolerance and 1.0% of optimization. Isolates that had < 80% similarity on the PFGE profiles were considered different types (Figure 1).

### Antimicrobial Susceptibility Test

The procedure for broth microdilution was adapted from the CLSI protocol for antimicrobial susceptibility testing (Clinical and Laboratory Standards Institute [CLSI], 2009). Antibiotic solutions were prepared in LAB susceptibility media (90% LSM and 10% MRS broth, adjusted to pH 6.7) (Klare et al., 2005). The following antibiotics were tested: ampicillin, chloramphenicol, clindamycin, erythromycin, fusidic acid, gentamycin, kanamycin, linezolid, streptomycin, teicoplanin, trimethoprim/sulfamethoxazole (1/19), and vancomycin (Sigma-Aldrich, St. Louis, MO, United States). The antibiotic susceptibility tests and their interpretations were carried out according to the CLSI guidelines (British Society for Antimicrobial Chemotherapy [BSAC], 2014; Clinical and Laboratory Standards Institute [CLSI], 2016).

### Pepsin Tolerance

A pepsin solution was prepared by suspending 3 mg/ml pepsin (Sigma-Aldrich, St. Louis, MO, United States) in a 0.85% sterile saline solution adjusted to pH 2.5. To test bacterial viability in the presence of pepsin, the 3 mg/ml pepsin solution, or saline at a pH of 7.0 as control, was inoculated with 10^6^ CFU/ml of *Lactobacillus* spp. and then incubated at 37°C for 4 h. The viable cell population was determined using the spread plate method. Each experiment was conducted in triplicate (Tokatli et al., 2015).

### Bile Salt Tolerance

The bile salt tolerance assay was performed as previously described (Jacobsen et al., 1999). In brief, each strain was adjusted to 1 × 106 CFU/ml in 0.3% Oxgall (Sigma-Aldrich, St. Louis, MO, United States) in PBS pH 7.3, or in PBS pH 7.3 alone as a control. Survival was tested at 37°C after 24 h by the spread plate method. Each experiment was conducted in triplicate.

### Cell-Free Supernatant (CFS) Preparation

Lactobacilli cell-free supernatants were cultured in MRS broth at 37°C for 24 h. The cultures were then centrifuged at 4000 rpm at 4°C for 30 min. The supernatants were sterilized by filtration through a 0.22 μm filter (Millipore, Billerica, MA, United States).

### Well Diffusion Assay

The agar well diffusion method (Tagg and McGiven, 1971) was modified to detect antimicrobial activities of supernatants isolated from *Lactobacillus* strains. First, MH agar plates were swabbed on the surface with CRE bacterial cultures. Then, 6 mm diameter wells were prepared and cell-free supernatants from isolated lactobacilli were loaded in the wells (100 μl/well). Following a 24-h incubation at 37°C, inhibition zones were recorded.

### Broth Microdilution Assay

Broth microdilution assay was carried out as previously described (Arena et al., 2016) with some modifications. Overnight cultures of pathogenic bacteria were inoculated into fresh MHB media and seeded into 96-well plates (BD Discovery Labware, Bedford, MA, United States). The CFS of each *Lactobacillus* culture was separated into three aliquots: The first aliquot received no treatment, the second aliquot was heated at 80°C for 10 min, and the third aliquot was neutralized to pH 7.0 with 1N NaOH. A 200 μl volume of test solution, consisting of 100 μl of the pathogenic bacterial culture (final inoculum was approximately 10^6^ CFU/ml) and 100 μl of one of the CFS aliquots, was mixed into the wells. The CFSs were diluted with MRS broth and used at different percentages (i.e., 10, 20, 30, 40, 50%) in the final 200 μl volume. The minimum inhibitory percentage (MIP), defined as the lowest percentage of supernatant that can inhibit the growth of pathogen, was monitored by measuring optical density (OD600 nm). The minimum bactericidal percentage (MBP) was defined as the lowest percentage of CFS that can kill all the pathogenic bacteria, as detected by subculturing treated samples onto MH agar. All tests were done in triplication.

### Time-Kill Test in Co-cultures

Carbapenem-resistant *Enterobacteriaceae* and *Lactobacillus* strains were individually cultured in their respective broth medium at 37°C for 24 h. The cultures were centrifuged at 6000 rpm, 22°C for 10 min to collect the cell pellet. Then, pathogenic bacteria were inoculated at 1 × 10^6^ CFU/ml and lactobacilli at 1 × 10^5^, 1 × 10^6^, 1 × 10^7^, or 1 × 10^8^ CFU/ml into mono-cultures or 1 × 10^6^ CFU/ml pathogenic bacteria co-culture with 1 × 10^5^, 1 × 10^6^, 1 × 10^7^, or 1 × 10^8^ CFU/ml lactobacilli in tubes containing 10 ml of MRS-MH broth (1:1) (Drago et al., 1997). Mono-cultures and co-cultures were incubated at 37°C for 48 h. Samples were collected at 0, 2, 4, 8, 24, and 48 h for the determination of viable cell count and pH measurements. A 1 ml aliquot of each sample was used to prepare serial dilutions that were poured onto the appropriate agar plates; MRS agar was used for *Lactobacillus* spp., while MacConkey Agar was used for *Enterobacteriaceae*. Plates were incubated at 37°C for 24 h and colonies were counted. The assay detection limit was 100 CFU/ml (Shah et al., 2016). All tests were done in triplication.

### Statistical Analysis

The paired *t*-test was used for statistical analysis. The level of significance for all analysis was *p* < 0.05.

## Results

### Microbiological Characteristics of *Lactobacillus* Isolates

A total of 57 *Lactobacillu*s isolates, including *L. plantarum* (*n* = 17), *L. paracasei* (*n* = 16), *L. rhamnosus* (*n* = 8), *L. fermentum* (*n* = 6), *L. casei* (*n* = 4), *L. brevis* (*n* = 2), *L. futsaii* (*n* = 2), *L. furfuricola* (*n* = 1), and *L. sakei* (*n* = 1) were collected for this study (Table 1). Based on the findings from MIC testing of these *Lactobacillu*s isolates, we determined that these isolates were highly susceptible to chloramphenicol, clindamycin, erythromycin, linezolid and streptomycin with susceptibility rates ranging from 91.2 to 100%. The resistance rates of these isolates against fusidic acid, kanamycin, trimethoprim/sulfamethoxazole, teicoplanin, and vancomycin ranged from 89.5 to 100% (Table 2).

**Table 1:** Name and number of each species among the 57 total *Lactobacillu*s isolates.

*Lactobacillus brevis* (*N* = 2)	LYC1152, LYC1113
*Lactobacillus casei* (*N* = 4)	LUC0095, LUC0123, LUC0197, LYC1229,
*Lactobacillus fermentum* (*N* = 6)	LUC0113, LUC0168, LUC0174, LUC0182, LUC0191, LYC1120
*Lactobacillus furfuricola* (*N* = 1)	LYC1039
*Lactobacillus futsaii* (*N* = 2)	LYC1037, LYC1038
*Lactobacillus paracasei* (*N* = 16)	LUC0018, LUC0040, LUC0044, LUC0048, LUC0097, LUC0180,
	LYC1119, LYC1142, LYC1149, LYC1151, LYC1154, LYC1156,
	LYC1162, LYC1164, LYC1235, LYC1237
*Lactobacillus plantarum* (*N* = 17)	LUC0125, LUC0128, LUC0219, LYC0289, LYC1031, LYC1088,
	LYC1112, LYC1115, LYC1117, LYC1138, LYC1141, LYC1143,
	LYC1144, LYC1146, LYC1159, LYC1303, LYC1322
*Lactobacillus rhamnosus* (*N* = 8)	LUC0103, LUC0103, LUC0115, LUC0127, LUC0192, LYC0413,
	LYC1065, LYC1118
*Lactobacillus sakei* (*N* = 1)	LYC1287

**Table 2:** Distribution of different antimicrobial agent MICs (mg/l) for 57 *Lactobacillus* isolates.

	≤0.06	0.12	0.25	0.5	1	2	4	8	16	32	64	128	>128
AMP	2	1		3	6	33	11	1					
CHL					1	5	46	5					
CLI	20	19	8	7	1	2							
ERY	33	22				1			1				
FA					2	2	1	1	7	13	2	15	14
GM				1	2	9	25	15	3	1	1		
KAN											14	26	17
LNZ					1	4	47	5					
STR						1	1	6	29	13	7		
TEC								1	1	3	2	5	45
SXT					2	4	1		1		1	10	38
VAN												1	56

*(1) SXT, only the MIC of trimethoprim is shown. (2) AMP: ampicillin; CHL, chloramphenicol; CLI, clindamycin; ERY, erythromycin; FA, fusidic acid; GM, gentamicin; KAN, kanamycin; LNZ, linezolid; STR, streptomycin; TEC, teicoplanin; SXT, trimethoprim/sulfamethoxazole (1/19); VAN, vancomycin.*

### *In vitro* Viability of *Lactobacillus* Isolates

Table 3 displays the results of the pepsin tolerance tests. The viability of 32 of the isolates was reduced by <2 log. Another 19 isolates were totally inhibited in these simulated conditions. Table 4 shows the results of the bile salt tolerance tests. With the exception of LYC1164, which had a 2.1-log reduction in growth, all of the isolates had a less than a 2-log reduction in cell viability after incubation in this simulated intestinal condition for 24 h. Based on the findings of these *in vitro* viability tests, we chose 31 *Lactobacillus* isolates with less than 2-log reductions in viability in both pepsin and bile salt tolerance tests. Because of their ability to survive in these simulated gastric and intestinal environments, we selected these strains for the assessment their antibacterial activity.

**Table 3:** Pepsin tolerance test results for 57 *Lactobacillus* isolates at pH 2.5 with 3.5 mg/ml pepsin.

*Lactobacillus* strain	Cell viability (log CFU/ml)		Reduction in cell viability (log)
	pH 7.0	pH 2.5, 3.5 mg/ml pepsin	
LUC0018	7.04 ± 0.08	4.54 ± 0.09	2.50
LUC0040	7.14 ± 0.07	7.01 ± 0.08	0.13
LUC0044	6.81 ± 0.00	4.59 ± 0.16	2.22
LUC0048	6.93 ± 0.04	4.50 ± 0.28	2.43
LUC0095	6.89 ± 0.02	0.00 ± 0.00	6.89
LUC0097	7.08 ± 0.03	4.60 ± 0.08	2.48
LUC0103	6.54 ± 0.00	0.00 ± 0.00	6.54
LUC0113	6.15 ± 0.00	0.00 ± 0.00	6.15
LUC0114	7.11 ± 0.00	0.00 ± 0.00	7.11
LUC0115	6.90 ± 0.08	0.00 ± 0.00	6.90
LUC0123	6.50 ± 0.14	0.00 ± 0.00	6.50
LUC0125	6.83 ± 0.18	0.00 ± 0.00	6.83
LUC0127	6.69 ± 0.21	5.89 ± 0.06	0.80
LUC0128	6.89 ± 0.06	0.00 ± 0.00	6.89
LUC0168	6.83 ± 0.25	6.68 ± 0.19	0.15
LUC0174	6.92 ± 0.15	6.81 ± 0.16	0.11
LUC0180	7.05 ± 0.10	6.60 ± 0.08	0.45
LUC0182	7.08 ± 0.08	6.90 ± 0.04	0.18
LUC0191	6.86 ± 0.06	6.36 ± 0.26	0.50
LUC0192	6.63 ± 0.04	0.00 ± 0.00	6.63
LUC0197	6.80 ± 0.02	0.00 ± 0.00	6.80
LUC0219	6.48 ± 0.25	6.23 ± 0.05	0.25
LYC0289	6.39 ± 0.12	4.85 ± 0.00	1.54
LYC0413	6.48 ± 0.00	6.11 ± 0.02	0.37
LYC1031	6.72 ± 0.03	5.09 ± 0.12	1.63
LYC1037	6.50 ± 0.14	0.00 ± 0.00	6.50
LYC1038	6.44 ± 0.06	0.00 ± 0.00	6.44
LYC1039	6.63 ± 0.04	0.00 ± 0.00	6.63
LYC1065	7.10 ± 0.05	6.89 ± 0.06	0.21
LYC1088	6.65 ± 0.00	6.50 ± 0.14	0.15
LYC1112	6.81 ± 0.10	6.59 ± 0.16	0.22
LYC1113	7.24 ± 0.01	4.93 ± 0.04	2.31
LYC1115	6.85 ± 0.21	6.54 ± 0.09	0.31
LYC1117	6.57 ± 0.12	6.24 ± 0.09	0.33
LYC1118	6.93 ± 0.04	6.77 ± 0.10	0.16
LYC1119	6.83 ± 0.02	6.54 ± 0.09	0.29
LYC1120	7.11 ± 0.02	6.68 ± 0.03	0.43
LYC1138	7.04 ± 0.03	6.95 ± 0.00	0.09
LYC1141	6.99 ± 0.05	6.70 ± 0.00	0.29
LYC1142	6.85 ± 0.11	6.81 ± 0.10	0.04
LYC1143	7.09 ± 0.06	6.94 ± 0.05	0.15
LYC1144	6.98 ± 0.06	0.00 ± 0.00	6.98
LYC1146	7.00 ± 0.03	6.03 ± 0.11	0.97
LYC1149	6.90 ± 0.12	6.44 ± 0.06	0.46
LYC1151	6.78 ± 0.05	6.20 ± 0.03	0.58
LYC1152	7.03 ± 0.04	5.15 ± 0.00	1.88
LYC1154	7.19 ± 0.04	6.89 ± 0.06	0.30
LYC1156	6.90 ± 0.08	3.60 ± 0.08	3.30
LYC1159	6.96 ± 0.08	6.88 ± 0.10	0.08
LYC1162	7.22 ± 0.02	0.00 ± 0.00	7.22
LYC1164	7.18 ± 0.04	6.81 ± 0.05	0.37
LYC1229	7.02 ± 0.06	6.96 ± 0.05	0.06
LYC1235	6.95 ± 0.10	0.00 ± 0.00	6.95
LYC1237	6.97 ± 0.02	0.00 ± 0.00	6.97
LYC1287	6.85 ± 0.11	0.00 ± 0.00	6.85
LYC1303	6.83 ± 0.02	0.00 ± 0.00	6.83
LYC1322	7.07 ± 0.01	5.72 ± 0.03	1.35

**Table 4:** Bile salt tolerance test results for 57 *Lactobacillus* isolates at pH 7.3 with 0.3% Oxgall.

*Lactobacillus* strain	Cell viability (log CFU/ml)		Reduction in cell viability (log)
	pH 7.3	pH 7.3, 0.3% Oxgall	
LUC0018	9.67 ± 0.10	8.62 ± 0.11	1.05
LUC0040	9.78 ± 0.18	8.60 ± 0.08	1.18
LUC0044	9.99 ± 0.02	8.48 ± 0.00	1.51
LUC0048	9.93 ± 0.07	8.79 ± 0.07	1.14
LUC0095	8.99 ± 0.08	8.08 ± 0.00	0.91
LUC0097	9.88 ± 0.10	8.67 ± 0.10	1.21
LUC0103	9.03 ± 0.14	8.41 ± 0.40	0.62
LUC0113	9.80 ± 0.02	8.97 ± 0.10	0.83
LUC0114	9.65 ± 0.00	9.06 ± 0.05	0.59
LUC0115	9.81 ± 0.00	9.00 ± 0.11	0.81
LUC0123	9.07 ± 0.20	8.41 ± 0.34	0.66
LUC0125	9.85 ± 0.00	8.38 ± 0.38	1.47
LUC0127	9.25 ± 0.07	8.44 ± 0.06	0.81
LUC0128	9.65 ± 0.00	8.65 ± 0.00	1.00
LUC0168	9.20 ± 0.14	8.83 ± 0.13	0.37
LUC0174	9.40 ± 0.00	9.14 ± 0.06	0.26
LUC0180	9.30 ± 0.00	8.70 ± 0.06	0.60
LUC0182	9.81 ± 0.16	9.13 ± 0.09	0.68
LUC0191	9.34 ± 0.45	8.88 ± 0.10	0.46
LUC0192	10.02 ± 0.00	8.85 ± 0.11	1.17
LUC0197	9.19 ± 0.16	8.30 ± 0.14	0.89
LUC0219	9.33 ± 0.20	8.99 ± 0.12	0.34
LYC0289	9.16 ± 0.02	8.11 ± 0.05	1.05
LYC0413	9.02 ± 0.00	8.59 ± 0.27	0.43
LYC1031	9.98 ± 0.00	8.88 ± 0.10	1.10
LYC1037	9.81 ± 0.00	9.11 ± 0.05	0.70
LYC1038	8.74 ± 0.06	6.99 ± 0.05	1.75
LYC1039	9.10 ± 0.06	8.55 ± 0.21	0.55
LYC1065	9.18 ± 0.00	8.87 ± 0.04	0.30
LYC1088	9.74 ± 0.00	9.09 ± 0.06	0.65
LYC1112	9.40 ± 0.00	8.88 ± 0.10	0.52
LYC1113	9.88 ± 0.00	9.36 ± 0.41	0.52
LYC1115	9.13 ± 0.01	8.13 ± 0.02	1.00
LYC1117	9.30 ± 0.25	8.41 ± 0.41	0.89
LYC1118	9.15 ± 0.21	8.94 ± 0.18	0.21
LYC1119	9.10 ± 0.17	8.44 ± 0.37	0.66
LYC1120	9.81 ± 0.00	8.88 ± 0.10	0.93
LYC1138	9.14 ± 0.06	8.37 ± 0.32	0.77
LYC1141	9.02 ± 0.20	8.68 ± 0.03	0.34
LYC1142	9.06 ± 0.12	8.26 ± 0.31	0.80
LYC1143	9.26 ± 0.19	8.76 ± 0.03	0.50
LYC1144	9.78 ± 0.00	9.06 ± 0.08	0.72
LYC1146	9.54 ± 0.00	8.47 ± 0.10	1.07
LYC1149	9.13 ± 0.00	8.08 ± 0.08	1.05
LYC1151	9.08 ± 0.09	8.65 ± 0.07	0.43
LYC1152	9.48 ± 0.00	9.02 ± 0.03	0.46
LYC1154	9.39 ± 0.30	8.78 ± 0.00	0.61
LYC1156	9.24 ± 0.23	8.36 ± 0.26	0.88
LYC1159	9.85 ± 0.00	8.04 ± 0.03	1.81
LYC1162	9.65 ± 0.00	8.48 ± 0.25	1.17
LYC1164	9.12 ± 0.06	7.02 ± 0.06	2.10
LYC1229	9.42 ± 0.17	8.45 ± 0.21	0.97
LYC1235	9.22 ± 0.24	8.71 ± 0.15	0.51
LYC1237	9.10 ± 0.02	8.94 ± 0.05	0.16
LYC1287	9.23 ± 0.05	8.05 ± 0.11	1.18
LYC1303	9.78 ± 0.00	8.74 ± 0.00	1.04
LYC1322	9.54 ± 0.00	8.12 ± 0.03	1.42

### The Results of the Agar Well Diffusion Method

**FIGURE 2:**
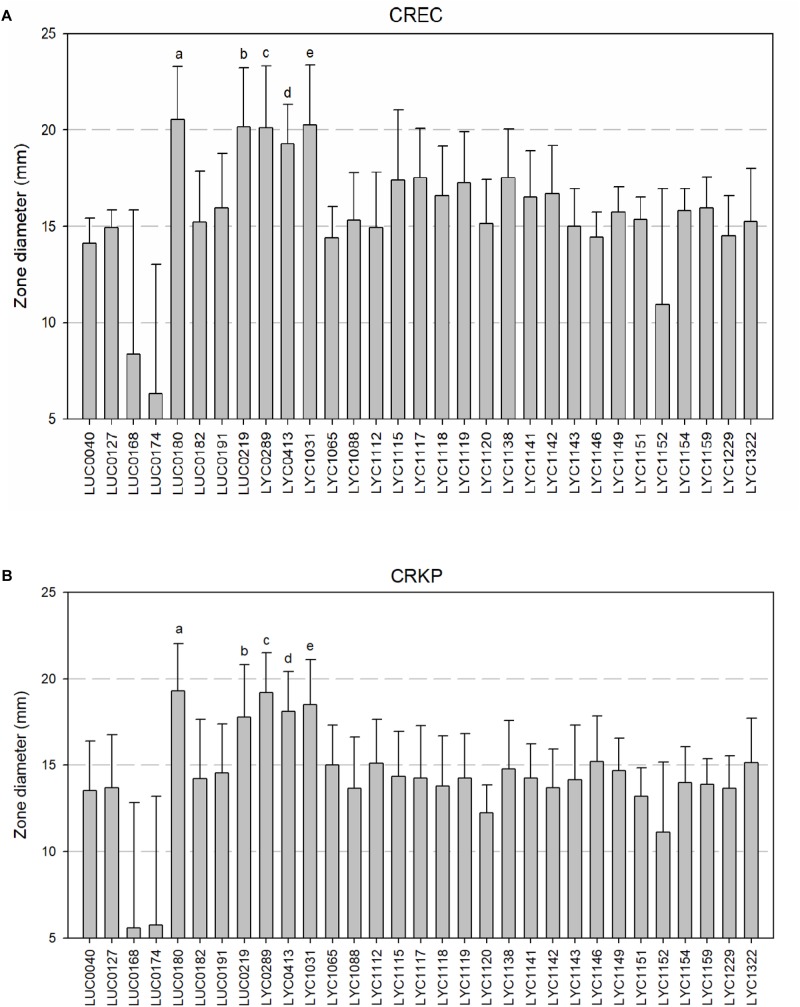
**(A)** Zone of inhibition of 31 *Lactobacillus* isolates against carbapenem-resistant *E. coli* by well diffusion assays. (a, b, c, d, e five isolates showed the significantly better activity than all of the other strains (all *p* < 0.05). However, there was no significant differences among them). **(B)** Zone of inhibition of 31 *Lactobacillus* isolates against carbapenem-resistant *K. pneumoniae* by well diffusion assays. [a, b, c, d, e five isolates showed the significantly better activity than all of the other strains (all *p* < 0.05). However, there was no significant differences among them].

The 21 isolates displayed the greatest activity against carbapenem-resistant *E. coli* with zones of inhibition greater than 15 mm (Figure 2A). The greatest activity against carbapenem-resistant *K. pneumoniae* isolates, with zones of inhibition greater than or equal to 15 mm, were observed in the nine isolates (Figure 2B). Overall, we chose five *Lactobacillus* strains (LUC0180, LUC0219, LYC0289, LYC0413, and LYC1031), which had the significantly better antibacterial activities than the most of the other strains (*p* < 0.05) as determined by the agar well diffusion and time-kill assay for further tests of antibacterial activity.

### The Results of MIP/MBP Tests

Table 5 shows the MIP and MBP for the five most potent *Lactobacillus* supernatants against CRE. The MIP of these five strains ranged from 10 to 30%. Except for LUC0180, which had an MBP of ≥40%, all of the strains had MBPs ranging from 20 – 40%. The inhibitory effects of these *Lactobacillus* strains did not change after heating up to 80°C for 10 min. However, the inhibitory effect disappeared once the pH increased to 7.0 (data not show). Additionally, the pH in the presence of LUC0180 seemed to be higher than the other four isolates.

**Table 5:** The resulting pH and MIPs/MBPs of five *Lactobacillus* strain supernatants (%) against carbapenem-resistant *E. coli* (CREC) and *K. pneumoniae* (CRKP).

CREC	pH	CRE78	CRE108	CRE128	CRE178	CRE202	CRE236	CRE240	CRE361	CRE387	CRE397
LUC0180	4.01	20/40	20/40	20/40	20/40	20/40	20/30	20/40	20/40	30/>50	30/>50
LUC0219	3.86	10/30	10/40	10/40	10/40	10/30	10/30	10/40	10/40	20/40	20/40
LYC0289	3.76	10/30	10/40	10/30	10/40	10/30	10/30	10/30	10/30	10/30	10/40
LYC0413	3.82	10/20	10/30	10/20	10/30	10/20	10/20	10/30	10/30	10/30	10/30
LYC1031	3.84	10/30	10/40	10/30	10/40	10/30	10/30	10/30	10/30	10/30	10/30

**CRKP**	**pH**	**CRE632**	**CRE716**	**CRE804**	**CRE825**	**CRE831**	**CRE851**	**CRE919**	**CRE929**	**CRE944**	**CRE945**

LUC0180	4.01	20/40	20/40	20/30	20/30	20/30	20/30	20/30	20/40	20/30	20/30
LUC0219	3.86	10/40	10/30	10/30	10/30	10/30	10/30	10/20	10/30	10/20	10/30
LYC0289	3.76	10/30	10/30	10/30	10/20	10/20	10/30	10/20	10/30	10/20	10/20
LYC0413	3.82	10/20	20/20	10/20	20/20	10/20	20/20	10/20	20/20	10/20	20/30
LYC1031	3.84	10/40	10/30	10/30	10/20	10/30	10/30	10/20	10/30	10/20	10/20

### The Results of Time-Kill Test

**FIGURE 3:**
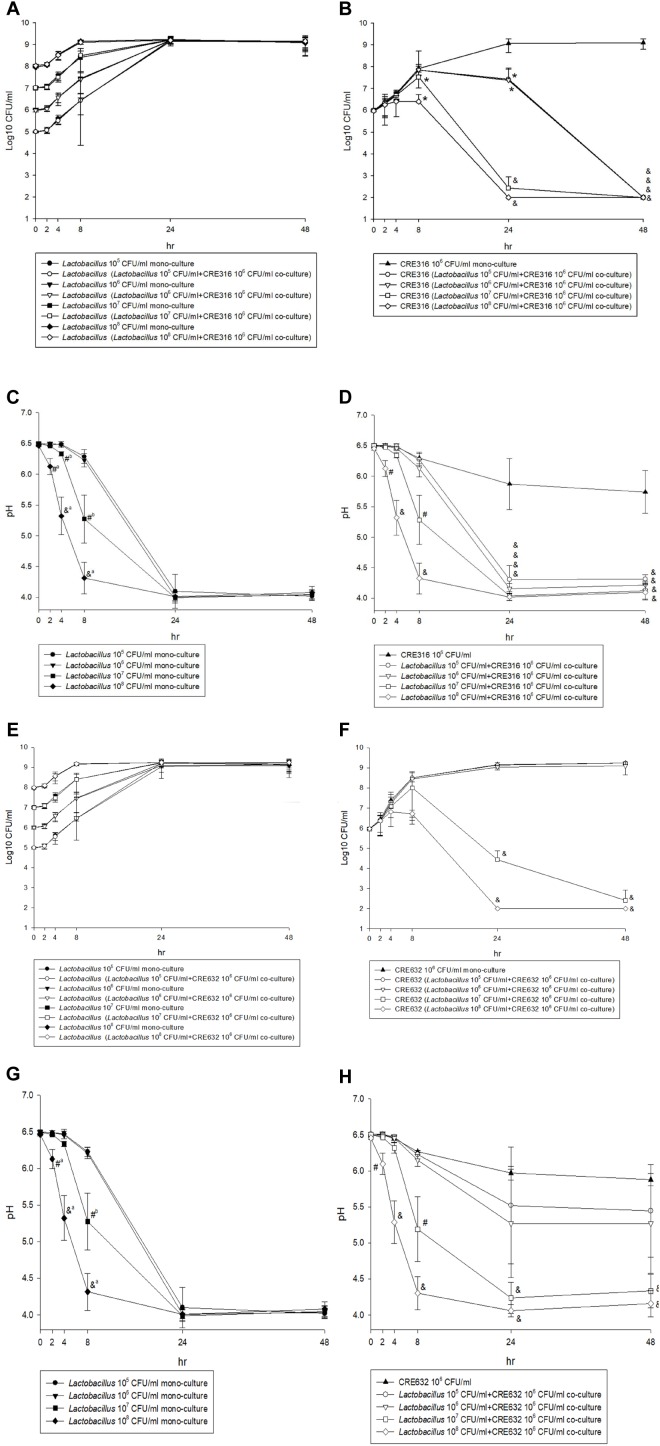
**(A,E)** The CFU change of five different concentration (10^5^ to 10^8^ CFU/ml) *Lactobacillus* isolates after mono-culture or co-culture with CRE316/ 632 for 48 h. **(B,F)** The CFU change of CRE316/ 632 after mono-culture or co-culture with five different concentration (10^5^ to 10^8^ CFU/ml) *Lactobacillus* isolates for 48 h and comparison between mono-culture and co-culture. **(C,G)** The pH changes of five different concentration (10^5^ to 10^8^ CFU/ml) *Lactobacillus* isolates mono-culture for 48 h. **(D,H)** The pH changes of CRE316/ 632 after mono-culture or co-culture with five different concentration (10^5^ to 10^8^ CFU/ml) *Lactobacillus* isolates and comparison between mono-culture and co-culture. (^a^ Means the comparisons between 10^8^ and 10^5,6,7^ CFU/ml lactobacillus isolates. ^b^ Means the comparisons between 10^7^ and 10^5,6^ CFU/ml *Lactobacillus* isolates. ∗*P*-value < 0.05, #*P*-value < 0.001, &*P*-value < 0.0001).

Figure 3 shows the results from time-killing test and assessment of the association between pH and antibacterial effects. After a 24-h incubation, there was no significant change between mono-cultures of lactobacilli and co-culture of lactobacilli and carbapenem-resistant *E. coli* (CRE316) in term of the concentration of lactobacilli (all *p* > 0.05) (Figure 3A). In contrast, the growth of CRE 316 was significantly inhibited after co-culture with different concentration lactobacilli after 24 h (all *p* < 0.05) and even 48 h (*p* < 0.0001) when comparing with mono-culture of CRE (Figure 3B). Furthermore, the decreases in pH were observed in the mono-cultures of lactobacilli,especially at the concentration of 10^8^ and 10^7^ CFU/ml (both *p* < 0.05) (Figure 3C). The decrease in pH remains significant while in the co-cultures with CRE316 compared with CRE316 monoculture (all *p* < 0.05, Figure 3D). The similar findings were also noted for the co-culture with carbapenem-resistant *K. pneumonia* (CR632) (Figure 3E–H).

## Discussion

In this study, five *Lactobacillus* strains, including three *L. plantarum* strains (LUC0219, LYC0289, LYC1031) and one each of *L. paracaseri* (LUC0180) and *L. rhamnosus* (LYC0413), exhibited good antibacterial activity against CREs. These inhibitory effects were demonstrated in through various tests including the agar well diffusion method, broth microdilution assay, and time-kill test. *Lactobacillus* spp. are non-pathogenic Gram-positive rods that are also recognized as normal human flora. Recently, several studies have reported that *Lactobacillus* strains can exhibit antibacterial activity through several mechanisms such as the production of antimicrobial substances or metabolites (hydrogen peroxide, lactic acid, bacteriocin), competition for nutrients, inhibition of bacterial adhesion to the mucosa, and enhancement of the immune response (Gill et al., 2001; Reid and Burton, 2002; Servin, 2004; Saulnier et al., 2009). Some *in vitro* studies have also shown that *Lactobacillus* strains can exhibit antimicrobial activity against *C. difficile*, *E. coli*, *Shigella* spp., *S. mutans, P. aeruginosa*, and *S. aureus* (Jamalifar et al., 2011; Mirnejad et al., 2013; Kumar et al., 2016; Kang et al., 2017; Ahn et al., 2018). To the best of our knowledge, this is the first study to document evidence of *Lactobacillus* antimicrobial activity against CRE. Although *in vitro* activity cannot be directly translated to *in vivo* effect, our findings suggest a promising role for *Lactobacillus* strains in the prevention and treatment of CRE colonization or infection. However, further animal studies are warranted to clarify this issue.

In the time-kill studies, the co-cultures with CREs did not influence the growth of lactobacilli. Additionally, effective inhibitory activity was generally observed at a pH of less than 4.2. These results were comparable with what we observed in our MIP/MBP experiments. By contrast, the antibacterial activity of *Lactobacillus* strains was dependent on an acidic environment and the inhibitory effect disappeared once the pH became greater than 6.5 during the MIP/MBP tests. Importantly, antimicrobial activity was not influenced by heating. This indicates that the inhibitory effect is mostly due to the acidic conditions, and not the production of bacteriocin-like substances. Similar findings were noted in the time-kill test. Previous reports have shown that the production of organic acids by probiotic organisms and the resulting decrease in culture pH is considered to be the principal antimicrobial effect (Zhang et al., 2011; Tejero-Sarinena et al., 2012). In addition to lowering the culture pH, Alakomi et al. (2000) reported that organic acids could also function as outer membrane permeabilizers of some gram-negative bacteria and enhance the activity of other antimicrobial metabolites. These findings suggest that acidic pH and/or the presence of organic acids may be essential for the antibacterial activity observed in the present study.

In this study, only 31 of the 57 *Lactobacillus* strains that were initially collected for assessment were found to be able to survive in the simulated gastric and intestinal environment. For clinical application, these characteristics are extremely important. To be functional in the lower enteric tract, the tolerance of lactobacilli to pepsin, low pH and bile salts is essential.

This study had several limitations. First, in addition to the effect of acid environment, we did not investigate the detail mechanism or molecular effectors of these function traits. Second, we used separated tests instead of a unique trait by using *in vitro* GIT system to evaluate the GIT resistance in this study. Further study is warranted to clarify these issues.

## Conclusion

Several *Lactobacillus* strains exhibit antibacterial activity against CRE. We suggest that this effect may have potential applications through the use of *Lactobacillus* strains as starter cultures in fermented foods or as food preservatives for controlling or preventing CRE infections. Additional studies are required to determine the effects of complex nutrients on the synthesis of the antibacterial substance, as well as to elucidate the mechanisms and genetic basis of the bactericidal activity. Further animal models are also necessary to document the *in vivo* survival of these acid, pepsin and bile salt tolerant lactobacilli in the lower enteric tract. If the same inhibitory effect can be documented *in vivo*, further studies including vancomycin-resistant *Enterococcus* should be performed in the future.

## Author Contributions

H-JT and Y-CL are the guarantors of this manuscript. C-CC, C-CL, H-LH, W-YH, H-ST, T-CW, and Y-CC contributed to the conception and design of the study. C-CC and H-JT analyzed and interpreted the data. C-CC, C-CL, and H-JT drafted the manuscript. All authors reviewed the manuscript.

## Conflict of Interest Statement

The authors declare that the research was conducted in the absence of any commercial or financial relationships that could be construed as a potential conflict of interest.
